# ZEB1 and Neural Stem Cells: Insights into Microglia-Conditioned Medium-Driven Neuroinflammation

**DOI:** 10.3390/cells14201587

**Published:** 2025-10-13

**Authors:** Elham Poonaki, Ulf Dietrich Kahlert, Walter Stummer, Sven G. Meuth, Ali Gorji

**Affiliations:** 1 Department of Neurology, Faculty of Medicine, Heinrich-Heine-University, 40225 Düsseldorf, Germany; elham.poonaki@ukmuenster.de (E.P.); svenguenther.meuth@med.uni-duesseldorf.de (S.G.M.); 2 Epilepsy Research Center, Münster University, 48149 Münster, Germany; 3 Molecular and Experimental Surgery, Faculty of Medicine, University Clinic for General-, Visceral-, Vascular- and Transplantation Surgery, Otto-Von-Guericke-University, 39120 Magdeburg, Germany; ulf.kahlert@med.ovgu.de; 4 Department of Neurosurgery, Münster University, 48149 Münster, Germany; walter.stummer@ukmuenster.de; 5 Shefa Neuroscience Research Center, Khatam Alanbia Hospital, Tehran 1996835911, Iran

**Keywords:** brain, neuroregeneration, neural stem cells, cell differentiation, multiple sclerosis

## Abstract

**Highlights:**

**What are the main findings?**

- ZEB1 knockdown in neural stem cells (NSCs) significantly reduced neurosphere formation, cell migration, reactive oxygen species generation, and cytokine produc-tion under both non-inflammatory conditions and inflammation induced by condi-tioned medium from LPS-activated microglia.
- ZEB1 is a regulator of NSC behavior, modulating their responses to a neuroinflam-matory milieu.

**What is the implication of the main finding?**

- Targeting ZEB1 may offer a novel therapeutic approach for controlling neuroinflam-mation and preserving neurogenesis in central nervous system disorders.
- Modulation of ZEB1 could help protect NSC function during inflammatory challenges.

**Abstract:**

Neuroinflammation is a key response to disturbed CNS homeostasis, largely mediated by activated microglia, and excessive microglia-driven inflammation can negatively impact neurogenesis. ZEB1 plays a crucial role in neurogenesis and brain development by influencing neural stem cell (NSC) maintenance, proliferation, and differentiation. This study aimed to evaluate how the knockdown of ZEB1 influences the behavior of NSCs in inflammatory environments. NSCs were isolated from the subventricular zone of rats, and ZEB1 knockdown was achieved using ZEB1 siRNA. A conditioned medium derived from lipopolysaccharide-activated microglia was utilized to induce inflammatory responses in NSCs. The silencing of ZEB1 in NSCs significantly reduced the expression of ZEB1. Furthermore, ZEB1 knockdown in NSCs resulted in a significant decrease in neurosphere formation, cell migration ability, reactive oxygen species generation, and various cytokine levels under both non-inflammatory and inflammatory conditions. These findings reveal the regulatory role of ZEB1 in the modulation of NSC behavior, suggesting that targeting ZEB1 may provide a potential therapeutic strategy for neuroinflammatory CNS disorders.

## 1. Introduction

Neuroinflammation is implicated in the pathophysiology of various neurodegenerative diseases, such as multiple sclerosis [1,2,3]. Inflammation inhibits both the generation of new neurons in the healthy state and the increase in neurogenesis that occurs after central nervous system (CNS) injury [4]. Neural stem cells (NSCs) have been investigated for their role in fostering brain repair in both acute and chronic inflammatory CNS conditions, offering a potential therapeutic approach through neuroprotection, immunomodulation, and tissue regeneration [5,6]. Under neuroinflammatory conditions, activated NSCs can proliferate, differentiate, and migrate to the injured site to replace damaged cells and promote structural and functional repair [7,8]. NSCs contribute to protecting neurons from inflammatory damage by secreting neuroprotective factors, maintaining and repairing the blood–brain barrier, and modulating pathways to either enhance or suppress inflammatory responses depending on the circumstances [9,10]. However, the transplantation of NSCs faces significant challenges, as both primary and pre-existing neuroinflammation, associated with compromised immunomodulatory effects, hamper the function and integration of NSCs [11]. Therefore, enhancing our understanding of how NSC responses are regulated under inflammatory conditions is crucial for developing effective therapeutic interventions [12,13].

Zinc Finger E-Box Binding Homeobox 1 (ZEB1), a transcription factor involved in gene regulation and cellular differentiation, plays a crucial role in both neurogenesis and the modulation of inflammatory processes across various pathological conditions [14,15,16]. ZEB1 is primarily known for its role in epithelial–mesenchymal transition (EMT), a process essential for cellular plasticity and implicated in various inflammatory processes [17,18]. ZEB1 exhibits a synergistic relationship with interleukin-6 (IL-6)/IL-11-STAT3 signaling, leading to the upregulation of the S100 protein pivotal in both EMT and inflammatory signaling pathways. In addition to acute inflammation, ZEB1 has been implicated in chronic inflammation, where it stimulates inflammatory cytokines along with reactive oxygen species (ROS) and reactive nitrogen species [19,20,21]. Microglia, the resident immune cells of the central nervous system, play a crucial role in mediating neuroinflammation associated with neurodegenerative diseases like multiple sclerosis and other neuroinflammatory conditions [22]. In this study, we explore the mechanisms of microglial activation under inflammatory conditions, utilizing conditioned medium from LPS-stimulated microglia to model their impact on neural environments. The present investigation is focused on examining the expression of ZEB1 in NSCs isolated from the subventricular zone (SVZ) of rat brains under both non-inflammatory and inflammatory conditions. Furthermore, we explored the impact of *ZEB1* gene knockdown in NSCs in these conditions.

## 2. Materials and Methods

### 2.1. NSC Culture

All experimental procedures were conducted in compliance with the National Institutes of Health Guide for the Care and Use of Laboratory Animals at the University of Münster, Germany (AZ:81-02.05.50.21.016/22.12.2021). To obtain the NSCs, adult rats (12–18 weeks) were anesthetized with isoflurane. Once the rats reached a surgical depth of anesthesia, they were decapitated to allow for the extraction of the brain under sterile conditions. The animals were monitored throughout the procedure to minimize distress. Primary NSCs were isolated from the SVZ of the Wistar rat brain (*n* = 5). After mechanical and enzymatic tissue digestion with trypsin/EDTA 0.25% (Sigma-Aldrich, Taufkirchen, Germany), single cells were cultured in Dulbecco’s Modified Eagle’s Medium/Nutrient Mixture F-12 (DMEM/F12; Sigma-Aldrich, Taufkirchen, Germany). The culture medium was prepared by combining 0.5% N2 supplement (Gibco^®^, Thermo Scientific, Waltham, MA, USA), 2 μg/mL heparin (Ratiopharm, Ulm, Germany), 1% L-glutamine (Sigma-Aldrich, Germany), 2% B27 supplement (Gibco^®^, Thermo Scientific, Waltham, MA, USA), 20 ng/mL epidermal growth factor (EGF; STEMCELL Technologies, Cologne, Germany), 10 ng/mL basic fibroblast growth factor (bFGF; STEMCELL Technologies, Cologne, Germany), and 1% penicillin/streptomycin (Sigma-Aldrich, Germany). After a cultivation period of 15–20 days, the cells were prepared for subsequent seeding. NSCs were passaged after forming spheres larger than 150 µm and were cultured in laminin-coating well plates for 3 to 5 days. The cells were incubated at 37 °C with 5% CO_2_. All experimental procedures were conducted in compliance with the National Institutes of Health Guide for the Care and Use of Laboratory Animals at the University of Münster, Germany. 

### 2.2. Primary Microglia Culture

Primary microglia were also isolated from neocortex of rat brains following the same age group as above-mentioned and then were dissected and exposed to trypsin/EDTA (0.25%, Sigma-Aldrich, Taufkirchen, Germany) for 5 to 10 min, and then neutralized with fetal bovine serum (FBS, Gibco^®^, Thermo Scientific, Waltham, MA, USA) and centrifuged at 900 rpm. Next, the cells were filtered through a 70 µm cell strainer and underwent another round of centrifugation at a speed of 1100 rpm. Subsequently, the cells were placed into the adherent 24-well plates to reach approximately 70% confluency per well. The culture medium was prepared with DMEM/F12 (Sigma-Aldrich, Taufkirchen, Germany) including 0.5% N2 supplement, 2 μg/mL heparin, 1% glutamine (Sigma-Aldrich, Taufkirchen, Germany), 2% B27 supplement, 20 ng/mL EGF, 10 ng/mL bFGF, and 1% penicillin/streptomycin (Sigma-Aldrich, Taufkirchen, Germany). The medium was changed after 24 h with the medium including DMEM/F12 (Sigma-Aldrich, Germany), 10% FBS, and 1% L-glutamine (Sigma-Aldrich, Taufkirchen, Germany). After 10 days, microglial cells were treated with 100 ng/mL lipopolysaccharides (LPS) from *Escherichia coli* O111:B4 (L2630, Sigma-Aldrich, Taufkirchen, Germany). Microglia were treated with LPS (100 ng/mL) for 16 h, after which the LPS-containing medium was discarded. Cells were then incubated with antibiotic-free NSC medium for 24–48 h to allow cytokine release. The supernatant was collected as CM, which was free of LPS. The morphological alteration of microglia was evaluated through quantitative assessment of amoeboid versus ramified cell populations. Furthermore, measurements of fluorescence intensity of microglia in both untreated and LPS-stimulated cultures were assessed by ImageJ (v1.53k; NIH, USA).

### 2.3. Cytotoxicity Assay

NSCs were cultured at a density of 2 × 10^4^ cells/well in 96-well plates and maintained at 37 °C for 72 h until reaching confluency (70–80%). The cells were then treated with 50% of the conditioned medium from LPS-activated microglia (CM) for either 24 or 48 h. After treatment, 100 μL of 10% WST-1 solution (11644807001, Sigma-Aldrich, Taufkirchen, Germany) was added to each well and incubated for 3 h. The WST formazan absorbance was measured at a wavelength of 440 nm by a microplate reader to evaluate the cell viability between CM-treated cells with the control group.

### 2.4. Apoptosis Assay Using Flow Cytometry

To investigate the potential of CM to induce apoptosis in NSCs, the Annexin V-DY-634/PI assay kit (ab214484, Abcam, Cambridge, UK) was used for flow cytometric analysis. NSCs from three different cells were seeded in a 24-well plate at a density of 8 × 10^4^ cells/well and subsequently treated with CM for durations of 24 and 48 h. Following treatment, the cells were washed twice with PBS and centrifuged at 1100 rpm for 5 min and then pooled for overall toxicity analysis. Following the initial step, 100 μL of 1X binding buffer was applied to the cells. This was followed by the addition of 5 μL of Annexin V-DY634 conjugate and 5 μL of propidium iodide staining solution. The mixture was then incubated in the dark at room temperature for 15 min. Subsequently, 400 μL of 1X binding buffer solution was added to each sample. A CytoFLEX™ flow cytometer was utilized for cell evaluation, and the data were analyzed using FlowJo version 10 software. Flow cytometry quantification was performed using annexin V/PI staining across biological replicates, analyzed with FlowJo software.

### 2.5. Transfection of ZEB1 siRNA

*ZEB1* siRNA transfection was performed using Lipofectamine^®^ 2000, following the manufacturer’s protocol (Thermo Fisher Scientific, Dreieich, Germany). The *ZEB1* sequence of the Silencer Select siRNAs (s130529, Ambion, Thermo Fisher Scientific, Dreieich, Germany) used in this experiment includes the sense strand: 5′-GGCUGUAGAUGGUAACAUATT-3′. Cells were seeded at a density of 8 × 10^4^ cells per well in 24-well plates and 2 × 10^4^ cells per well in 96-well plates, depending on the experimental requirements. Then, the cells were incubated for 72 h at 37 °C in the presence of 5% CO_2_ to reach a confluency level of 60–70%. Following this, the cells were transfected with the reagent according to the manufacturer’s guidelines. For transfection, 15 pmol of *ZEB1* siRNA was used for cells in 96-well plates, and 30 pmol was used for cells in 24-well plates. The Silencer Select Negative Control siRNA (#4390843, Ambion, Thermo Fisher Scientific, Dreieich, Germany) was used as a control. The siRNA was diluted in 50 µL of Gibco™ Opti-MEM™ and incubated for 5 min. It was then combined with 1 µL of Lipofectamine^®^ 2000 (11668027, Invitrogen, Thermo Fisher Scientific, Dreieich, Germany) diluted in 50 µL of Opti-MEM (Gibco^®^, Thermo Scientific, Waltham, MA, USA) and incubated for an additional 5 min at room temperature. The mixture of the two dilutions was then incubated for 20 min. To achieve optimal transfection efficacy, all steps were performed in a penicillin/streptomycin-free condition. The resulting siRNA mixture was then added to the cell culture medium and maintained at 37 °C for an additional 48 h regarding our post-efficacy assessment at 24, 48, and 72 h using immunocytochemistry (ICC) and fluorescence microscopy (Keyence, Osaka, Japan). The transfection efficiency was determined by the ratio of fluorescent to non-fluorescent cells using ImageJ software.

### 2.6. ICC Assay

ICC assay was conducted following a previously established procedure [23] to assess the characteristics of cells at different stages of NSC differentiation and the effects of *ZEB1* siRNA under inflammatory conditions. NSCs were grown on laminin-coated 24-well plates at a density of 8 × 10^4^ cells/well transfected with Lipofectamine^®^ 2000 and *ZEB1* siRNA as mentioned. Two groups were also exposed to CM regarding the experimental groups for 48 h. The cells were fixed with paraformaldehyde 4% (Sigma-Aldrich, Taufkirchen, Germany). After exposure of cells to 0.2% Triton for 15 min at room temperature, the cells were treated with a blocking buffer including goat serum in dark conditions at room temperature for 30 min. Nestin (ab254048, Abcam, Cambridge, UK) 1:500, GFAP (ab279291, Abcam, Cambridge, UK) 1:500, and SOX2 (ab79351, Abcam, Cambridge, UK) 1:500 as a marker of NSCs derived from SVZ as well as anti-ZEB1 (SAB5700810, Sigma-Alderich, Taufkirchen, Germany) and IBA1 (019-19741, FUJIFILM Wako Pure Chemical Corp., Osaka, Japan) 1:500 for microglial cells were used as primary antibodies. The plates were incubated overnight at 4 °C. In the next step, the cells were washed with PBS and were then exposed for two h to goat anti-mouse IgG (Abcam, Cambridge, UK) or goat anti-rabbit IgG (Abcam, Cambridge, UK) labeled with FITC based on the host of every antibody used as the secondary antibody. Cells were imaged using fluorescence microscopy (Keyence, Osaka, Japan). ICC images were quantified using ImageJ software for marker intensity and cell counts.

### 2.7. Colony Formation Assay

To assess the behavior of ZEB1-regulated stem cells in an inflammatory environment, a laminin-coated 96-well plate was used for the colony formation assay. Single cells (2 × 10^4^/well) were seeded on the laminin-coated 96-well plates, and treated with *ZEB1* siRNA and/or CM. The experiment was observed at predetermined intervals (1, 3, 7, 10, and 14 days). The images were captured utilizing an inverted microscope (Keyence, Osaka, Japan) and evaluated by ImageJ software.

### 2.8. Wound Healing Assay

A wound-healing approach was used to assess the migration ability of cells. In this study, we investigated the role of ZEB1 in NSC migration using a wound-healing approach (*n* = 6). A sterile 100 μL pipette tip was utilized to create a scratch in NSC cultured in 24-well plates with confluency between 85% and 95%. Subsequently, debris and non-adherent cells were removed using PBS (D6796, Sigma-Aldrich, Germany). After that, CM and *ZEB1* siRNA were applied to the cells. Wound closure in each group was evaluated at 0, 24, and 48 h using an inverted microscope and analyzed by ImageJ software.

### 2.9. Quantitative Polymerase Chain Reaction (qPCR) Assessment

Total RNA was obtained from treated stem cells after 48 h in 24-well plates (8 × 10^4^ cells/well and triplicate) using the QIAGEN RNA Purification Kit, following the manufacturer’s recommendations. Subsequently, the High-Capacity cDNA Reverse Transcription Kit (QIAGEN, Hilden, Germany) was employed to convert the extracted RNA into complementary DNA (cDNA), following the specified protocols. Quantitative PCR analysis was performed using a Real-Time PCR system (CFX Connect^®^, Bio-RAD, Feldkirchen, Germany) along with specific primers for ZEB1, IL6, IL-1β, IL17, TNF-α, and NFkB (Table 1). Gene expression levels were assessed via the Light Cycler 96 qPCR system (BioRAD, Germany), with GAPDH and β-Actin as internal control genes. The relative gene expression levels were quantified through the delta-delta Ct method (2^–∆∆Ct^ method).

### 2.10. ROS Assessment

To evaluate the ROS expression, the NSCs were cultivated in a 96-well plate coated with laminin (*n* = 3). The wells were then treated with *ZEB1* siRNA and/or CM. After that, cells were washed with 1X buffer and maintained in the dark at 37 °C for 30 min using 100 μL of a 20 μM H2DCFDA (ab113851, Abcam, Cambridge, UK) solution based on the manufacturer’s protocol. The fluorescent microscope (Keyence, Osaka, Japan) was used to visualize, and images were analyzed by ImageJ software.

### 2.11. Statistical Analyses

The data were analyzed and visualized using Prism 8.0.1 (GraphPad, GraphPad Software, San Diego, CA, USA). Data are presented as box-and-whisker plots. In each plot, the horizontal line denotes the median. The box boundaries correspond to the first and third quartiles, while the whiskers extend to the minimum and maximum values of the dataset. Individual observations are shown as dots. Statistical analysis was conducted using one-way ANOVA for ICC and qPCR assays, and two-way ANOVA for colony formation, wound healing, and ROS assays. A *p*-value of less than 0.05 was considered statistically significant.

## 3. Results

### 3.1. NSCs Characterization

**Figure 1:**
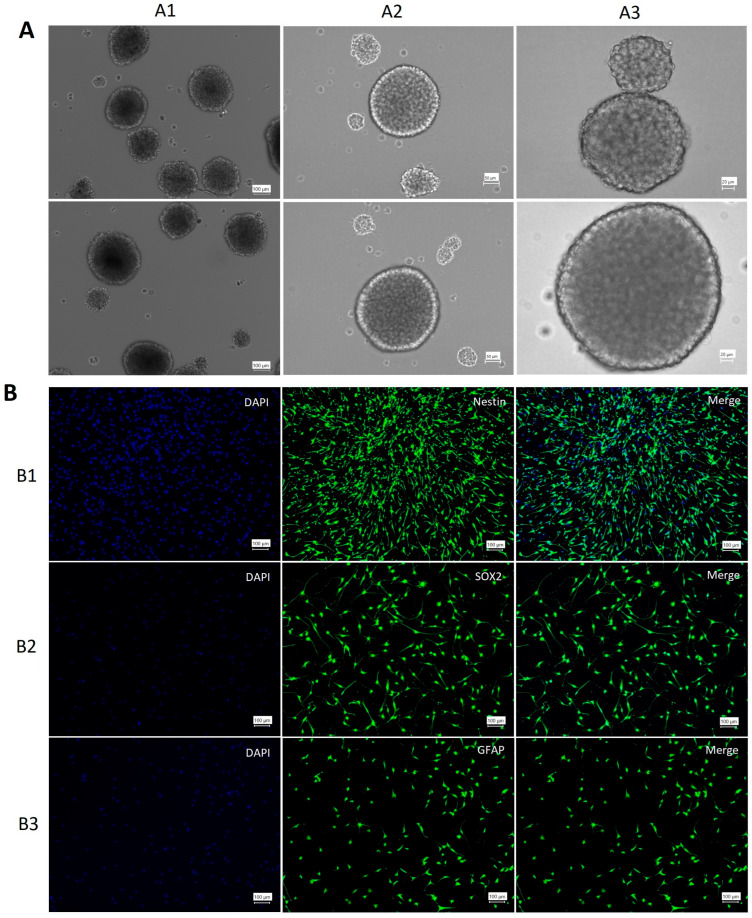
Characterization of neural stem cells. (**A**) 1–3: Spheres of NSCs in different dimensions. (**B**) Characterization of NSCs. The expression of nestin (B1), SOX2 (B2), and GFAP (B3) confirmed the presence of NSCs in the culture medium. DAPI (blue) stained the nucleus, and GFP (green) indicated antibody expression.

The clonal formation of neurospheres is an indicator of NSC self-renewal. After a 10–15-day incubation period, neurospheres were observed (Figure 1A). ICC was conducted using anti-nestin, anti-GFAP, and anti-SOX2 antibodies to confirm the presence of NSCs. The results demonstrated that NSCs extracted from the SVZ expressed nestin, SOX2, and GFAP (Figure 1B).

### 3.2. Microglial Activation and NSC Inflammatory Response

**Figure 2:**
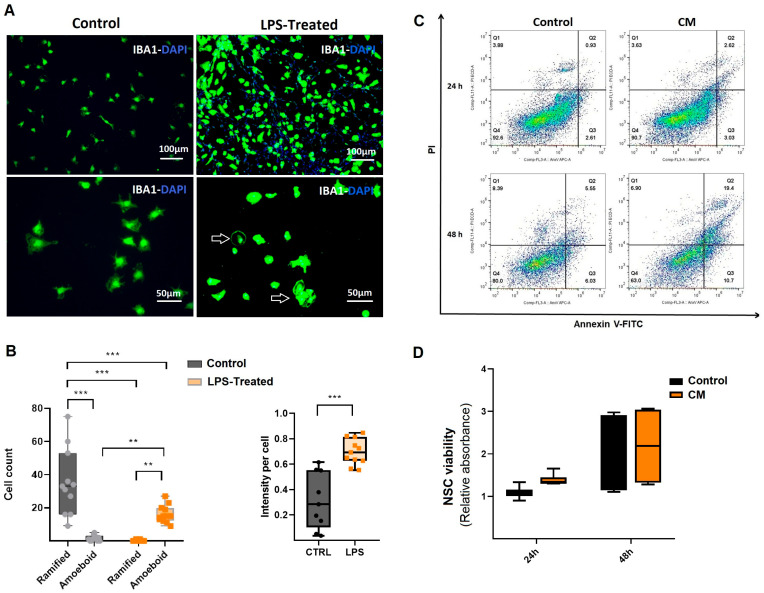
Evaluation of conditioned medium derived from microglial cells. (**A**) Morphology and characterization of activated microglial cells. Microglial characterization was performed using the IBA1 antibody. Amoeboid-shaped cells indicated microglial activation in response to LPS treatment. Images are shown at different magnifications. (**B**) Compared to the ramified (non-activated) microglia in the control group, LPS exposure significantly increased both fluorescence intensity and the proportion of amoeboid microglia (*n* = 3). (**C**) A single flow cytometry analysis of three various NSCs treated with conditioned medium from LPS-activated microglia (CM) for 24 and 48 h. Apoptosis was assessed using Annexin-PI double staining, with untreated NSCs as the control group. Our findings showed that early apoptosis increased in CM-treated NSCs after 48 h. (**D**) WST viability assay of NSCs treated with CM. Results indicated that CM treatment did not induce cytotoxic effects in NSCs compared to the control groups (*n* = 3). ** *p* ≤ 0.01; *** *p* ≤ 0.001.

To induce an inflammatory response, rat microglial cells were stimulated with LPS [24,25,26]. The effects of LPS stimulation on microglia were assessed using ICC. Microglial cells were characterized using the IBA1 marker, which revealed morphological changes following LPS stimulation. ICC analysis indicated the activation of microglia, characterized by amoeboid morphology, which includes a larger soma and a reduction in ramified processes [27] (Figure 2A). This morphological shift was further validated through quantitative assessment of amoeboid versus ramified cell populations, alongside measurements of fluorescence intensity in both untreated and LPS-stimulated microglial cultures (Figure 2B). The analysis revealed a significant increase in fluorescence intensity as well as in the ratio of amoeboid cells compared to the control group (*p* ≤ 0.001; Figure 2B). Following microglial treatment with LPS, CM (the inflammatory medium) was collected and used to induce inflammation in NSCs. A single flow cytometry analysis at 24 h from these three various NSCs, performed for qualitative assessment, revealed no significant early apoptosis or necrosis. However, by 48 h, there was an increase in early apoptosis, suggesting that NSCs exhibit a time-dependent, regulated inflammatory response to CM (Figure 2C). Furthermore, WST viability assays confirmed that CM treatment did not induce significant cytotoxicity in NSCs at either 24 or 48 h compared to the untreated control group (Figure 2D). These findings demonstrate that LPS application activates microglial cells, and the CM derived from these activated cells induces inflammation in NSCs while preserving their viability.

### 3.3. Evaluation of ZEB1 Expression in NSCs

The *ZEB1* gene knockdown was carried out through the utilization of *ZEB1* siRNA was delivered by Lipofectamine^®^ 2000 (see M+M section). The study included four experimental groups: (*i*) a non-treated control group, (*ii*) a group with *ZEB1* gene silencing using specific siRNA (*ZEB1*-siRNA), (*iii*) a group treated with CM, and (*iv*) a group treated with both *ZEB1*-siRNA and CM (*ZEB1*-siRNA + CM).

**Figure 3:**
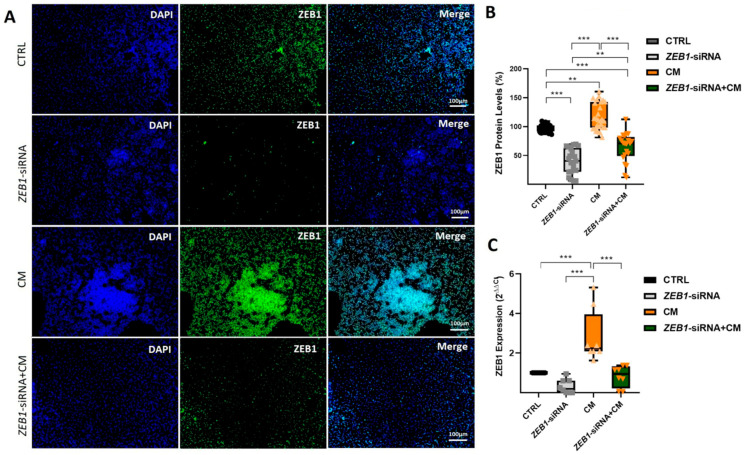
Assessment of ZEB1 expression in experimental treatment groups. (**A**) Immunocytochemistry (ICC) images showing ZEB1 expression in different neural stem cell (NSC) groups (*n* = 5) following siRNA-mediated knockdown and/or treatment with conditioned medium from LPS-activated microglia (CM). ZEB1 expression was visualized using green fluorescent protein (GFP), while nuclei were counterstained with DAPI (blue). (**B**) Quantitative analysis of ICC images using ImageJ software. (**C**) Quantitative PCR analysis of ZEB1 expression in different NSC groups after siRNA-mediated knockdown and/or subsequent treatment with CM. The control group serves as the baseline reference, with its relative expression value normalized to 1. These data indicate the modulatory role of ZEB1 in regulating the NSC response to inflammatory processes. ** *p* ≤ 0.01; *** *p* ≤ 0.001.

ZEB1 expression was assessed in various treatment groups using ICC and qPCR following 48 h of exposure to CM and *ZEB1* silencing. ICC analysis revealed a significant reduction in ZEB1 expression in the *ZEB1*-siRNA group compared to the control group, confirming the effective silencing of *ZEB1* (*p* ≤ 0.001; Figure 3A,B). Conversely, NSCs exposed to CM alone exhibited a significant increase in ZEB1 expression compared to the control group (*p* ≤ 0.01), as well as to the *ZEB1*-siRNA and *ZEB1*-siRNA + CM groups (*p* ≤ 0.001) (Figure 3A,B). Furthermore, ZEB1 expression was significantly higher in the *ZEB1*-siRNA + CM group compared to the *ZEB1*-siRNA group (*p* ≤ 0.01; Figure 3A,B). Moreover, PCR analysis revealed a significant upregulation of *ZEB1* in NSCs treated with CM compared to all other groups (*p* ≤ 0.001; Figure 3A,C). These results indicate that *ZEB1* silencing via siRNA effectively modulates the CM-induced upregulation of ZEB1 expression in NSCs.

### 3.4. Modulatory Effect of ZEB1 on NSC Colony Formation

**Figure 4:**
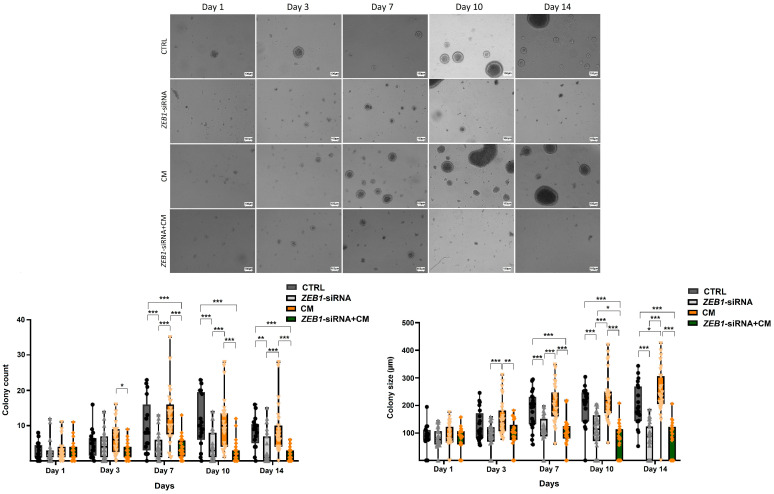
Effect of *ZEB1* knockdown on colony formation. Analysis over 14 days revealed distinct effects of *ZEB1* knockdown and conditioned medium from LPS-activated microglia (CM) on colony formation. A significant reduction in both colony size and number was observed in the *ZEB1* knockdown groups compared to the control and CM groups (*n* = 6). These findings revealed the distinct impacts of *ZEB1* knockdown and CM on colony dynamics, indicating that ZEB1 potentially regulates NSC colony formation. * *p* ≤ 0.05; ** *p* ≤ 0.01; *** *p* ≤ 0.001. Scale bar 100 μm.

Analysis of colony formation across different groups revealed a significant increase in colony number in the CM group compared to the ZEB1-siRNA + CM group after 3 days of cell treatment with CM and siRNA (*p* ≤ 0.05). Furthermore, the colony size in the CM group was significantly larger than the ZEB1-siRNA + CM group (*n* = 6; *p* ≤ 0.01) and the ZEB1-siRNA group (*p* ≤ 0.001) after 3 days of NSC treatment. After seven days, a significant increase in colony number was observed in the CM group compared to the ZEB1-siRNA group (*p* ≤ 0.001). Similarly, the control group exhibited greater colony number and size than both *ZEB1*-siRNA and *ZEB1*-siRNA + CM groups after 7 days (*p* ≤ 0.001). Moreover, after 10 days of NSC culture, colony size in the *ZEB1*-siRNA group was significantly larger than in the *ZEB1*-siRNA + CM group (*p*  ≤  0.05). After 14 days, the CM group displayed larger colonies compared to the control group (*p*  ≤  0.05). These findings indicate the differential effects of *ZEB1* knockdown and CM on colony dynamics over time. Furthermore, our data suggest that *ZEB1* knockdown plays a regulatory role in NSC colony formation in both non-inflammatory and inflammatory conditions (Figure 4).

### 3.5. The Impact of ZEB1 on NSC Migration

**Figure 5:**
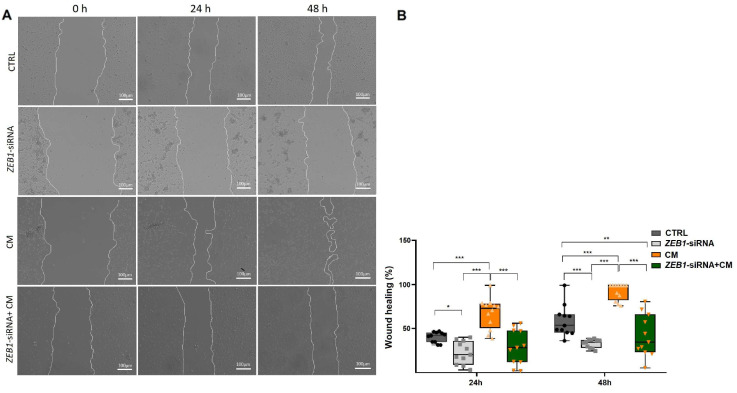
*ZEB1* silencing impairs NSC migration in the wound healing assay. (**A**,**B**) Wound healing assay analysis at 24 and 48 h revealed significantly enhanced wound closure in NSCs treated with conditioned medium from LPS-activated microglia (CM) compared to other groups at both time points (*n* = 6). In contrast, *ZEB1* silencing in NSCs significantly reduced wound closure compared to the control and CM groups, indicating impaired migratory capacity. * *p* ≤ 0.05, ** *p* ≤ 0.01, and *** *p* ≤ 0.001.

NSC migration is a fundamental process in brain development and repair, ensuring proper neuronal positioning and the potential for regeneration following injury [28]. Our results revealed that treatment with CM alone significantly accelerated wound closure compared to other groups at both 24 and 48 h (*p* ≤ 0.001; Figure 5A,B). Conversely, NSCs treated with ZEB1-siRNA exhibited a significantly reduced migration rate compared to the control group at 24 h (*p* ≤ 0.05) and 48 h (*p* ≤ 0.001) (Figure 5A,B). The ZEB1-siRNA + CM group exhibited a significantly lower wound closure rate than the control group only after 48 h (*p* ≤ 0.01; Figure 5A,B). These findings point to the role of ZEB1 in regulating NSC migration, suggesting its potential involvement in neural repair and regeneration.

### 3.6. The Impact of ZEB1 on Cytokine Levels

**Figure 6:**
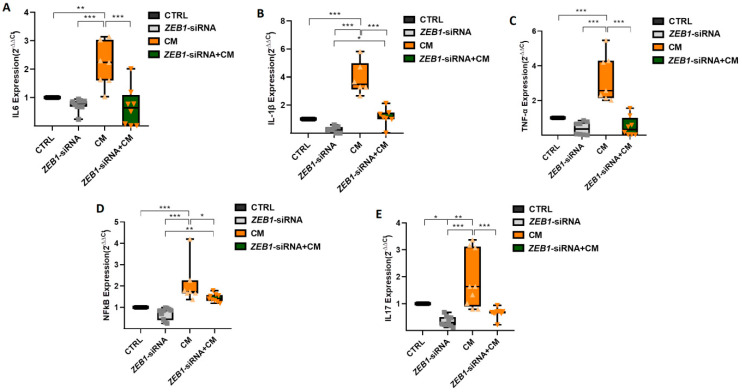
Impact of *ZEB1* silencing on cytokine expression in NSCs. The expression levels of IL-6 (**A**), IL-1β (**B**), TNF-α (**C**), NFκB (**D**), and IL-17 (**E**) were analyzed using qPCR (*n* = 5). Application of CM resulted in the enhancement of all cytokines compared to the control group. *ZEB1* silencing resulted in a significant decrease in the expression of these inflammatory cytokines in the *ZEB1*-siRNA + CM group, indicating that ZEB1 plays a critical role in regulating neuroinflammation. The control group serves as the baseline reference, with its relative expression value normalized to 1. * *p* ≤ 0.05, ** *p* ≤ 0.01, and *** *p* ≤ 0.001.

The qPCR assay was conducted to investigate the expression levels of four key cytokines, IL-6, IL-1β, TNF-α, and NFκB, as well as the Th17-associated cytokine IL-17. The results showed no significant difference in the expression of IL-6, IL-1β, TNF-α, and NFκB between the *ZEB1*-siRNA and control groups. However, treatment with CM significantly upregulated the expression of IL-6 (*p* ≤ 0.01), IL-1β (*p* ≤ 0.001), TNF-α (*p* ≤ 0.001), NFκB (*p* ≤ 0.001), and IL-17 (*p* ≤ 0.01) in NSCs compared to both the *ZEB1*-siRNA and control groups (Figure 6A–E). Moreover, cytokine expression was significantly lower in the *ZEB1*-siRNA + CM group compared to the CM-only group, with significant reductions in IL-6 (*p* ≤ 0.001), IL-1β (*p* ≤ 0.001), TNF-α (*p* ≤ 0.001), NFκB (*p* ≤ 0.05), and IL-17 (*p* ≤ 0.001). Furthermore, the expression levels of IL-1β (*p* ≤ 0.05) and NFκB (*p* ≤ 0.01) were significantly higher in the *ZEB1*-siRNA + CM group compared to the *ZEB1*-siRNA group (*p* ≤ 0.001). These findings suggest that CM promotes an inflammatory response in NSCs, while *ZEB1* silencing modulates cytokine expression, potentially influencing neuroinflammatory processes.

### 3.7. The Impact of ZEB1 on ROS

**Figure 7:**
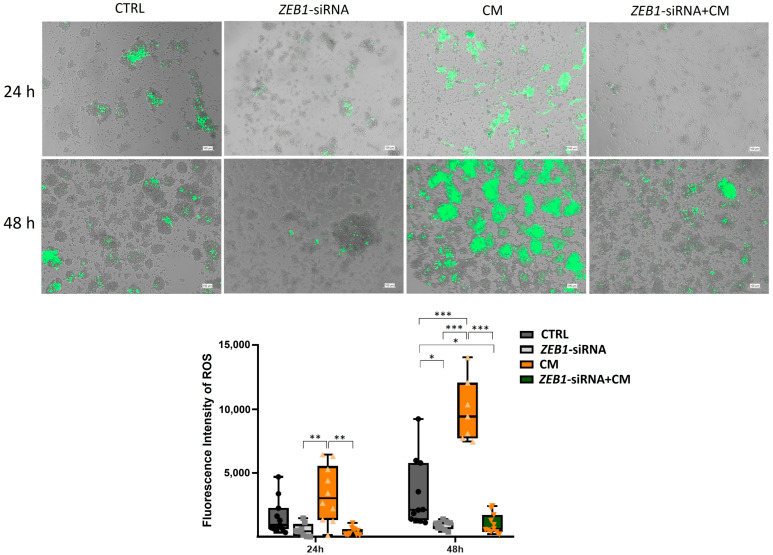
Effects of *ZEB1* silencing and CM on reactive oxygen species (ROS) generation in neural stem cells (NSCs). The fluorescent marker H2DCFDA was utilized to detect ROS in NSCs. Analysis at 24 and 48 h revealed a significant increase in ROS values in CM-treated groups. NSCs with *ZEB1* silencing exhibited markedly lower ROS levels, even under inflammatory conditions, compared to both the CM-treated and control groups, with the most pronounced difference observed at 48 h (*n* = 3). * *p*  ≤  0.05, ** *p*  ≤  0.01, and *** *p*  ≤  0.001.

Our results demonstrated that treatment with CM significantly elevated ROS production in NSCs compared to the control group after 48 h (*p* ≤ 0.001). Moreover, ROS levels in the CM-treated group were significantly higher than those in the *ZEB1*-siRNA and *ZEB1*-siRNA + CM groups at both 24 h (*p* ≤ 0.01) and 48 h (*p* ≤ 0.001). After 48 h, ROS levels in the control group were significantly higher than those in the *ZEB1*-siRNA and *ZEB1*-siRNA + CM groups (*p* ≤ 0.05). These findings suggest that CM enhances oxidative stress in NSCs, while *ZEB1* silencing reduces ROS generation, potentially influencing cellular stress responses (Figure 7).

## 4. Discussion

This study examined the role of ZEB1 in regulating neuroinflammatory responses in NSCs induced by CM obtained from LPS-activated microglia. Our findings reveal that ZEB1 is integral in maintaining NSC viability, migration, and colony formation, as well as in modulating oxidative stress in both physiological states and inflammatory conditions. Moreover, ZEB1 regulates key cytokine expression, indicating its essential role in the equilibrium of the neuroinflammatory milieu and supporting NSC homeostasis. Previous studies align with our findings, demonstrating that CM from LPS-activated microglia induces the release of pro-inflammatory cytokines (such as TNF-α, IL-1β, IL-6, IL-17, NFκB), promotes the generation of ROS, enhances apoptosis, and disrupts cell viability, migration, and colony formation [29,30,31,32,33,34]. We extracted conditioned medium from LPS-activated microglial cells isolated from the neocortex of rat brains to simulate the inflammatory environment observed in neurodegenerative disorders such as MS. Microglia are pivotal in triggering inflammation in response to LPS exposure and hypoxic conditions. Although these stimuli come from different pathological contexts, they activate similar molecular pathways, including oxidative stress and the production of pro-inflammatory cytokines like IL-1β, TNF-α, and IL-6. This common inflammatory profile suggests that the activation of microglia is linked to neuronal damage in both neurodegenerative diseases and CNS injuries associated with hypoxia, highlighting a potential shared mechanism underlying diverse brain pathologies [35,36,37,38].

Several studies have investigated the role of ZEB1 in physiological conditions, indicating its high expression in NSCs, where it is essential for maintaining stemness, self-renewal, differentiation, migration, and neurodevelopmental processes [15,16,39,40]. Our in vitro studies revealed the significant impact of the inflammatory microenvironment on the expression of ZEB1 as well as its effect on NSC characteristics and behavior. ZEB1 and inflammation are dynamically interconnected in a reciprocal regulatory loop within epithelial and tumor cells, where pro-inflammatory cytokines, such as TGF-β, enhance ZEB1 expression under inflammatory conditions [14]. This occurs via the activation of key signaling pathways, including Smad, tyrosine kinase receptors, NF-κB, and JAK1–STAT3 [41]. In turn, ZEB1 is involved in maintaining immune cell viability, mobility, and cytokine production. It modulates the expression of key cytokines, including IL-1β, IL-6, IL-8, IL-17, and TNF-α, by regulating the TGF-β-related STAT3 signaling pathway and NF-κB activity [42,43,44,45]. Furthermore, previous studies revealed that ZEB1 dynamically regulates gene expression by either activating or repressing key inflammatory mediators, such as Il-1β and TNF-α, and contributes to inflammation and cell death [46]. The reduction in ZEB1 disrupts the cancer cell-macrophage positive feedback loop, decreasing inflammation and oxidative stress, and ultimately preventing tumor progression [19]. ZEB1 suppression in T-cells alleviates the development and severity of symptoms in an animal model of autoimmune inflammatory diseases, while the downregulation of ZEB1 reduces pathogenic cytokine expression in T-cells from patients with multiple sclerosis [44]. The present findings reveal a bidirectional relationship between ZEB1 and inflammation in NSCs, pointing to its essential role in maintaining immune balance in NSCs. Inflammatory insults upregulate ZEB1 expression, while ZEB1, in turn, regulates cytokine production. This is evidenced by the significantly increased cytokine secretion and ROS generation in NSCs under inflammatory conditions, which is effectively disrupted by ZEB1 knockdown.

Effective neurogenesis needs a balanced transition from NSC proliferation to differentiation, ensuring that the generated cells migrate and integrate at the optimal time for proper CNS development and function [47,48]. ZEB1 plays a pivotal role in neurogenesis by the regulation of neural progenitor cell exit from the SVZ, neuronal differentiation, and migration via the modulation of distinct gene networks [16]. ZEB1 depletion disrupts the delicate equilibrium and coordinated timing between radial glial cell proliferation and differentiation, leading to aberrant neuronal migration and impaired glial differentiation [49]. Moreover, ZEB1 is essential in the tangential migration of un-polarized cerebellar granule neuron progenitors, while its downregulation is pivotal for their transition into mature, polarized cerebellar granule neurons and providing appropriate cerebellar development [50]. Our findings highlight ZEB1 as a key modulator of NSC proliferation and migration under both physiological and inflammatory conditions. Our study exposure to microglial CM increased apoptotic signaling without a corresponding reduction in overall cell viability. Microglial CM increased apoptotic signaling without reducing overall NSC viability, likely reflecting the detection of early apoptotic events before loss of metabolic activity and the possibility that only a subpopulation of cells is affected [51].

The ZEB1–inflammation axis plays an important role in the progression of various neuroinflammatory disorders by fostering cell proliferation and migration, promoting a pro-inflammatory microenvironment, impairing microglial and astrocyte function, and compromising the integrity of the blood–brain barrier [14,52]. NSCs offer an encouraging therapeutic approach for neuroinflammatory disorders, with their capacity for self-renewal, multi-lineage differentiation, micromilieu modulation, and bioactive factor secretion, while genetic modification further enhances these properties by promoting regenerative potential [53]. The role of ZEB1 in the modulation of NSC function, particularly under neuroinflammatory conditions, makes it an attractive target for NSC-based therapies in various neurological disorders, such as multiple sclerosis, traumatic brain injury, and neurodegenerative diseases. Modulation of ZEB1 expression in NSCs can enhance their resilience to inflammatory conditions, thus optimizing their therapeutic potential for tissue repair. The reduction in neuroinflammation through ZEB1 deactivation reduces key inflammatory mediators, thereby promoting a more neuroprotective and regenerative environment [54]. Consequently, targeting ZEB1 in NSC therapy could thus optimize therapeutic outcomes by achieving a balance between neuroprotection, inflammation, and tissue regeneration. Future research should investigate the downstream molecular mechanisms by which ZEB1 regulates inflammatory responses, including whether it directly controls cytokine gene expression or modulates other signaling pathways and transcription factors. Furthermore, in vitro and in vivo investigations are required to elucidate the exact role of ZEB1 in regulating the balance between anti-inflammatory responses and cell proliferation/survival of NSCs in neuroinflammatory CNS disorders, with a particular focus on neuroprotection and tissue regeneration.

## Figures and Tables

**Table 1:** Primer Sequences for rat genes utilized in qPCR analysis.

Gene	Stream	Sequence (5′-3′)
ZEB1	Forward	GCCCATCGAGCTTCCTGTAA
	Reverse	TGGAAACATGGCTCCTGTGC
IL-1β	Forward	GGGTGGTTCAAGGCATAACA
	Reverse	GTCGAGATGCTGCTGTGAGA
IL6	Forward	TCCTACCCCAACTTCCAATGCTC
	Reverse	TTGGATGGTCTTGGTCCTTAGCC
IL17	Forward	GGGAAGTTGGACCACCACAT
	Reverse	CGCCTTCTTTTCAGGGTGGA
NFkB	Forward	ACACAGGACCAGGGACAG
	Reverse	AGGGGTTGTTGTTGGTCTGG
TNF-α	Forward	AAATGGGCTCCCTCTCATCAGTTC
	Reverse	TCTGCTTGGTGGTTTGCTACGAC

## Data Availability

The data presented in this study are available on request from the corresponding author.
